# Reverse Engineering the Yeast RNR1 Transcriptional Control System

**DOI:** 10.1371/journal.pone.0013895

**Published:** 2010-11-16

**Authors:** Grace Mao, James P. Brody

**Affiliations:** Department of Biomedical Engineering, Henry Samueli School of Engineering, University of California Irvine, Irvine, California, United States of America; University of Maryland-Baltimore, United States of America

## Abstract

Transcription is controlled by multi-protein complexes binding to short non-coding regions of genomic DNA. These complexes interact combinatorially. A major goal of modern biology is to provide simple models that predict this complex behavior. The yeast gene RNR1 is transcribed periodically during the cell cycle. Here, we present a pilot study to demonstrate a new method of deciphering the logic behind transcriptional regulation. We took regular samples from cell cycle synchronized cultures of *Saccharomyces cerevisiae* and extracted nuclear protein. We tested these samples to measure the amount of protein that bound to seven different 16 base pair sequences of DNA that have been previously identified as protein binding locations in the promoter of the RNR1 gene. These tests were performed using surface plasmon resonance. We found that the surface plasmon resonance signals showed significant variation throughout the cell cycle. We correlated the protein binding data with previously published mRNA expression data and interpreted this to show that transcription requires protein bound to a particular site and either five different sites or one additional sites. We conclude that this demonstrates the feasibility of this approach to decipher the combinatorial logic of transcription.

## Introduction

Proteins binding to short, specific DNA sequences can regulate gene expression. These proteins, called transcription factors, enhance or repress transcription. Transcription factor binding sites are generally short (less than 12 base pairs) in length and are usually located in the promoter region of the regulated gene. In the simplest case, the binding of a single protein to the gene's promoter can enhance or repress expression. In more complex cases, expression is regulated through a combination of multi-protein complexes binding to several distinct elements. The determination of the location and decoding of the combinatorial logic of all these regulatory elements would provide an important annotation to the complete genome sequence and could lead to a better understanding of development and evolution [1]–[4].

Deciphering the transcriptional regulatory code is a central challenge of modern biomedical research. Years of research have shown that cellular differentiation is mostly governed through regulatory control of transcription within each cell [2]. Thus deciphering this code will lead to a better understanding of cellular differentiation.

Several different assays have been applied to this problem. DNAse I protection mapping can be used to locate the binding sites of specific proteins on DNA or to identify locations where crude fractions of protein bind [5], [6]. Protein binding microarrays have produced comprehensive binding data for hundreds of different DNA binding proteins [7]–[10]. Chromatin immunoprecipitation is a powerful technique to identify, across the genome, sequences that are bound to specific transcription factors [11]–[16].

The different approaches to the problem have been synthesized into comprehensive identification of regulatory elements in the yeast genome [17] and for parts of the human genome by the NHGRI ENCODE project [18], [19]. These projects have led to mass identification of regulatory sites, but they do not provide any information on how these regulatory sites interact—the regulatory program.

Deciphering the regulatory program requires many measurements of binding between nuclear protein and specific DNA sequence. Neither protein binding microarrays nor chromosome immunoprecipitation can provide such measurements. The critical barrier to deciphering transcriptional control programs is the accumulation of data on nuclear protein binding to specific DNA sequences and resulting mRNA levels. Our approach to overcoming this barrier is to develop a surface plasmon resonance based assay [20]–[23].

Previously, we demonstrated that one could identify regulatory elements using surface plasmon resonance [24]. We did this by showing a significant change in SPR signal correlated with both nuclear protein binding to DNA sequence representing a particular regulatory element *and* an increased level of promoter activity. We also demonstrated that we can *monitor* dynamic changes in the occupancy of regulatory elements by monitoring yeast nuclear protein binding to a region of the RNR1 promoter as the cell cycle progresses [25].

**Figure 1:**
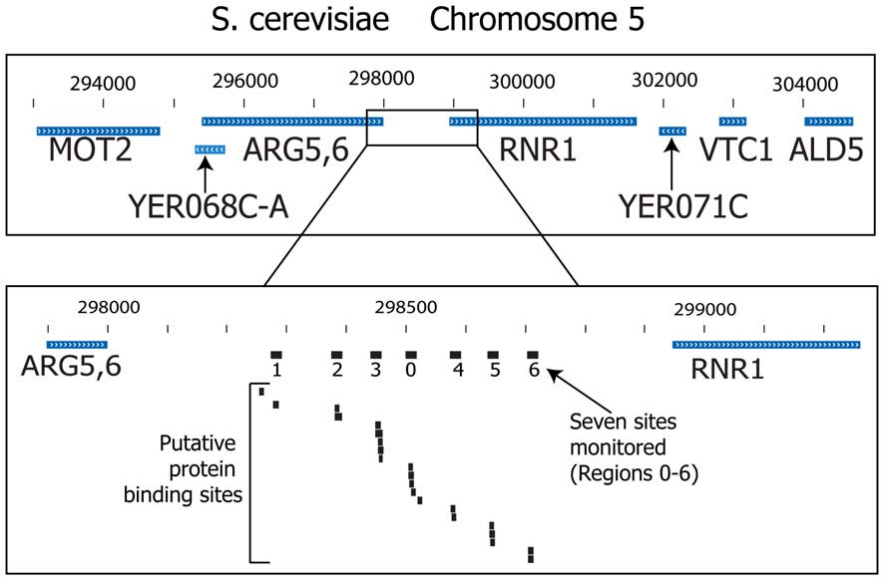
We monitored the binding of nuclear protein to seven different 16 bp regions of the RNR1 promoter, as shown in this figure. The top shows the general region of Chromosome 5, while the bottom focuses specifically on the region between the coding sequences for ARG5,6 and RNR1. We labeled the seven sites monitored as Regions 0 through Region 6 or R0–R6. Each of the seven contained one or more sites previously identified as a putative protein binding site [17]. The image was generated by the UCSC genome browser [40].

Here we extend our previous work on one region of the RNR1 promoter to six other regions. These seven encompass most of the putative protein binding sites in the RNR1 promoter identified by a comprehensive, multi-pronged approach [17], as shown in Figure 1. Analysis of these seven regions allows for the determination of putative regulatory control systems.

Surface plasmon resonance sensors have previously been applied to nucleic acid/protein studies. Much of this work has focused on measuring kinetic rates between purified protein and short stretches of DNA [20], [22], [26], [27]. Surface plasmon resonance was used to characterize the interactions between human estrogen receptors and estrogen response elements [23]. A novel nanostructure based sensor was used to detect interactions between a nucleic acid aptamer and thrombin protein [28]. Aptamer/protein studies were performed with a novel PDMS microfluidic surface plasmon resonance imaging system [29]. A recent novel application used an SPR sensor to test whether specific transcription factors bind anywhere on an entire promoter (1,000–3,000 bp) [30].

## Results

*RNR1* lies on yeast chromosome 5, see Figure 1. About seven different regions have been identified as likely transcriptional regulatory sites [17]. Like most yeast genes, *RNR1* has a compact and well characterized promoter. Thus it presents an ideal case for testing the ability of this assay to decipher its transcriptional regulatory program.

DNA (each 16 bp long) representing these seven regions were synthesized and separately attached to a surface plasmon resonant sensor. We treated a yeast culture to synchronize their cell cycles, took samples from the culture every 15 minutes, and purified nuclear protein from the samples. We measured the amount of nuclear protein binding to the seven different regions.

**Figure 2:**
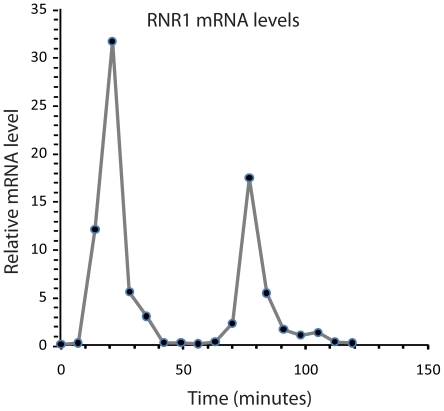
Relative *RNR1* (YER070W) mRNA levels as a function of time after alpha factor release (beginning of G1 phase of the cell cycle). This data was measured by others [31] with DNA microarrays.

*RNR1* mRNA levels were taken from published yeast microarray data [31]. These were collected following alpha factor arrest and are presented in Figure 2. Spellman *et. al* used DNA microarrays to comprehensively estimate relative mRNA levels of all yeast genes at 18 time points across the cell cycle [31]. They found that RNR1 mRNA levels reached two relative maximums, a first at about 21 minutes after synchronization, and a second at about 77 minutes [31].

### Nuclear protein binding to different regions of the RNR1 promoter

**Figure 3:**
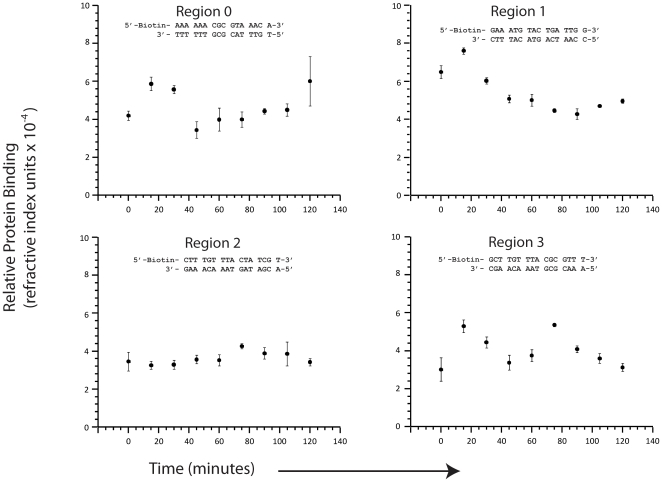
Surface plasmon measurements of nuclear protein binding to four different 16 bp long regions of the *RNR1* promoter. Each measurement was repeated three times and the data points represent the mean value, while the error bars indicate three times the standard deviation in the mean.

**Figure 4:**
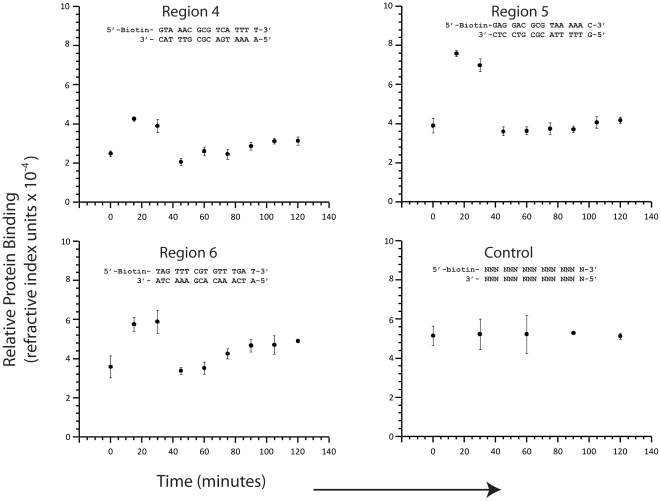
Surface plasmon measurements of nuclear protein binding to three different 16 bp long regions of the *RNR1* promoter and a random control 16 bp. Each measurement was repeated three times and the data points represent the mean value, while the error bars indicate three times the standard deviation in the mean. No significant differences were measured in nuclear protein binding to the control.

After we established a synchronous yeast cell culture, we extracted nuclear protein at 15 minute intervals and measured the relative amount of nuclear protein that bound to the seven different 16 bp regions of the RNR1 promoter listed in Figure 1. Each measurement was repeated three times to provide error estimates. The results are presented in Figures 3 and 4.

### Control experiments

Two types of control experiments were performed. First, we extracted nuclear protein from unsynchronized yeast cells and measured the relative nuclear protein binding of this sample to each of the seven different regions of the RNR1 promoter. This provided a baseline with which to compare the binding fro synchronized cells.

Second, we immobilized degenerate 16 bp DNA (NNN NNN NNN NNN NNN N) (where N can be any of the four common nucleotides) to the sensor surface. This degenerate DNA was synthesized with equal molar concentrations of each base (A, C, G, and T) at each location. We measured how much nuclear protein, extracted at different time points, bound to this sequence. Since this degenerate DNA consists of many different (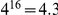 billion) sequences, we expect it to bind to many different proteins, which will average out and not show any variation with the cell cycle. As expected, we found no significant change in the amount of protein bound to this degenerate DNA at each time point.

**Figure 5:**
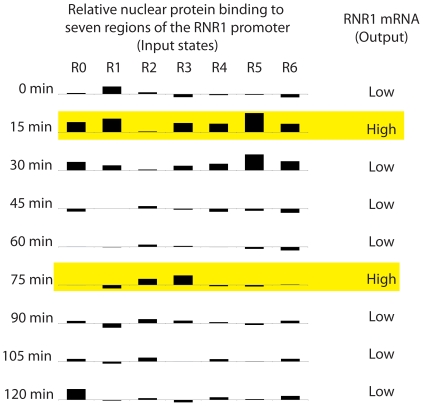
This diagram summarizes control of RNR1 transcription. The amount of nuclear protein binding to seven different regions of the RNR1 promoter is shown as black bars, with the height of the bars proportional to the difference between the binding measured at from nuclear protein at the given time point and binding measured with nuclear protein extracted from unsynchronized cells. On the right, the RNR1 mRNA levels are summarized. The left can be thought of as the input states, while the right represents the output.

**Figure 6:**
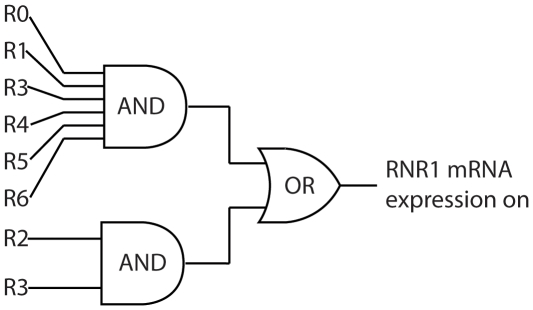
This digital circuit diagram represents the inferred logic governing RNR1 mRNA expression. High levels of expression occur if nuclear protein is bound to regions 0, 1, 3, 4, 5, and 6 or if nuclear protein is bound to regions 2 and 3.

We collated this data into a “promoter profile”, as shown in Figure 5. This figure graphically depicts how proteins are binding and releasing from the targeted regions of *RNR1*'s promoter. (Levels are depicted relative to unsynchronized cells, so in some cases these levels are negative.) It also summarizes the mRNA data (Figure 2) as either on/off or high/low. This digitization of the data, both the nuclear protein/DNA binding (input states) and the *RNR1* mRNA levels (output states), allows us to suggest the regulatory program encoded into the DNA. Our suggestion is shown in Figure 6 as a digital circuit. Using standard notation, it could be equivalently written as,
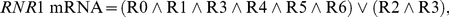
(1)indicating that *RNR1* mRNA only results if protein complexes are bound to regions 2 and 3, or to regions 0, 1, 3, 4, 5, and 6.

This result suggests a hypothesis about how different regulatory elements interact to regulate transcription of *RNR1*. The hypothesis is generated from a correlation analysis of multiple observations. A rigorous test of the hypothesis could be performed by directly altering key regions of DNA, for instance deleting Region 3. The significance of this approach is that one can generate these hypotheses in a rapid, high throughput manner. Furthermore, the hypothesis could itself be tested through the accumulation of more data.

## Discussion

This assay has some limits. It only identifies transcriptional regulation. Protein levels are regulated at many different points (e.g. transcription, translation, histones, ubiquitin-proteasome degradation). However, most regulation is thought to occur at the level of transcription [32].

The assay can not specifically identify protein complexes bound to the DNA. Three parameters could be measured to better identify these protein complexes: first, the kinetic binding constants between the protein and DNA [25], second, binding to specific antibodies [33], and third, the molecular weight of the complex, which is related to the increase in surface plasmon resonance signal. Each of these three factors should be dependent upon the identity of the protein complex. The measurement of all three, along with the knowledge that the protein binds to a specific DNA sequence should allow one to uniquely identify the protein complex.

This assay can be implemented as a higher throughput assay. The development of surface plasmon resonance imaging instruments [34]–[36] allow one to immobilize many different sequences 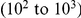 of DNA onto a surface and simultaneously accumulate measurements of nuclear lysate binding to these different regions. This provides substantial improvement in throughput, when hundreds of targets must be tested. Surface plasmon resonance imagers can measure several hundred interactions simultaneously at the level of about 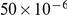 RIU. This is sensitive enough to measure the targeted interaction, in this work we measured the interaction as typically several times greater than 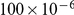 RIU.

In conclusion, we applied a novel method, surface plasmon resonance analysis of nuclear protein/DNA binding, to decipher how different regulatory elements interact to regulate transcription of a single gene, *RNR1*.

## Materials and Methods

### Yeast cell cultures and synchronization

As previously reported [25], we used the PY1 strain of *Saccharomyces cerevisiae* (yeast) [37]. Yeast cultures were grown in YPD medium (2% yeast extract, 4% peptone, and 4% dextrose) at 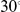
C with a temperature controlled heater and shaker. Cultures were grown for approximately 20 hours until cells reached late-log phase. Following [31], yeast cells were synchronized by adding 12 ng/ml of 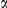-factor to culture for 3 hours. This 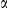-factor was subsequently removed by twice washing the cells, replacing the supernatant with fresh medium, and re-suspending the cells each time [25]. Samples were taken at regular intervals after establishing synchronous cultures and then processed to extract nuclear protein. Synchronization was confirmed by flow cytometry [25].

### Nuclear protein extraction

We extracted nuclear protein from each sample of the synchronous culture, as previously described [25]. Briefly, yeast cells were converted to spheroplasts by digesting the cell walls. Spheroplasts were lysed, centrifugation (13,000 rpm for 30 min) separated the cellular lysate from the nuclear material. The nuclear material was further purified by gradient centrifugation and dialysis. Protease activity was inhibited by a cocktail of protease inhibitors added to the extraction buffer.

The concentration of nuclear protein was determined using the Bradford assay [38]. Nuclear extract from each sample was normalized to a concentration of 0.33 mg/ml in protein binding buffer (20 mM Hepes (pH 7.6), 10 mM MgSO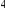, 1 mM EGTA, 20% glycerol, 75 mM ammonium sulfate).

### Surface plasmon resonance measurements

Surface plasmon resonance measurements were made with the Spreeta SPR sensor. The experimental set up includes a data acquisition and control computer, a syringe pump, and a Spreeta evaluation kit. The three channel Spreeta evaluation kit consists of several Spreeta sensor modules, a three channel flow cell, an electronic controller with comprehensive software, and an integrated flow block. The sensor modules are made by Sensata; other components are made by Nomadics.

The flow block was used to connect the Spreeta sensor module with the control box and to secure the flow cell to the surface of the sensor. The flow cell provides three independent flow channels. Each channel is approximately 4.5 mm long and 0.1 mm wide. The flow cell confines solution to the narrow channels, which correspond to the sensor surface.

The sensor data was analyzed to determine relative protein binding by measuring the difference in steady-state refractive index level before and after the addition of nuclear protein. Each experiment was repeated three times to provide error estimates.

### Attaching DNA to the gold surface

Double stranded DNA representing seven different regions of yeast chromosome 5, (see Table 1), was attached to the sensor surface using bovine serum albumin (BSA) as an intermediate. First, two complementary single-stranded DNA fragments, derivatized with biotin at the 5′ end, (see Table 1) each at 450 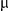
M concentration, were added together into a microtube. The microtube was placed into boiling water and allowed to slowly cool to room temperature. This annealing process produces double stranded DNA.

**Table 1:** Oligonucleotides used.

Control		5′-biotin-	NNN NNN NNN NNN NNN N -3′
		3′-	NNN NNN NNN NNN NNN N -5′
Region 0	R0	5′-Biotin-	AAA AAA CGC GTA AAC A -3′
		3′-	TTT TTT GCG CAT TTG T -5′
Region 1	R1	5′-Biotin-	GAA ATG TAC TGA TTG G -3′
		3′-	CTT TAC ATG ACT AAC C -5′
Region 2	R2	5′-Biotin-	CTT TGT TTA CTA TCG T -3′
		3′-	GAA ACA AAT GAT AGC A -5′
Region 3	R3	5′-Biotin-	GCT TGT TTA CGC GTT T -3′
		3′-	CGA ACA AAT GCG CAA A -5′
Region 4	R4	5′-Biotin-	GTA AAC GCG TCA TTT T -3′
		3′-	CAT TTG CGC AGT AAA A -5′
Region 5	R5	5′-Biotin-	GAG GAC GCG TAA AAA C -3′
		3′-	CTC CTG CGC ATT TTT G -5′
Region 6	R6	5′-Biotin-	TAG TTT CGT GTT TGA T -3′
		3′-	ATC AAA GCA CAA ACT A -5′

These oligonucleotides were chosen to represent known protein binding sites in the RNR promoter. Each contains a biotin on the 5′ end of the strand, which is used to immobilize the strand to the surface. The control oligonucleotides are synthesized such that the bases A, C, G, and T occur with equal probability at each site.

The immobilization scheme was implemented by flowing different solutions across the sensor surface. The sensor was monitored to confirm the appropriate surface modifications took place. The solutions contained (in order) biotin-BSA (0.67 mg/ml), streptavidin (0.33 mg/ml) and biotin-DNA (450 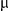
M) in PBS (1.37 mM NaCl, 2.7 mM KCl, 4.3 mM Na
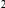
HPO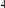, 1.4 mM KH
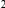
PO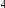 at pH 7.3), which is also the running buffer. These were stored at 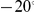
C, and thawed before use. Changes in refractive index adjacent to the sensing surface were monitored. Solutions remained in contact with the sensing surface until a stable refractive index value was reached, indicating the binding is at equilibrium. The running buffer was injected between each solution to remove non specifically bound molecules.

We previously measured the DNA surface density to be 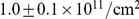 [39]. Using this surface density, we estimate an average spacing of about 30 nm between DNA molecules on the surface. This is much greater than the diameter of the DNA binding proteins (about 5 nm). Steric hindrance is not an issue.

### Measuring nuclear extract binding to DNA

The nuclear extract (0.33 mg/ml of protein) in protein binding buffer (20 mM HEPES (pH 7.6), 10 mM MgSO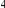, 1 mM EGTA, 20% glycerol, 75 mM ammonium sulfate) flowed across the sensor. The nuclear extract was stored at 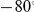
C to prevent any degradation. Binding buffer was then injected to remove any non-specifically bound protein.

### Cleaning

To restore the surface of the sensor to its original state, it was gently wiped with a Kimwipe wet by 6 N HCl and then flushed with water. This procedure was repeated three times. Then, 70% ethanol was used to wipe the surface followed by flushing with water; this was repeated three times. This cleaning procedure effectively removed all the immobilized layers. This was confirmed by measuring the refractive index of pure water as 1.3330. After each experiment was done, all syringes and tubes were rinsed thoroughly by water three times.
